# Knowledge and attitudes of U.S. medical students regarding the care of Asian American patients: a cross-sectional survey study

**DOI:** 10.1186/s12909-021-02568-0

**Published:** 2021-03-06

**Authors:** Sharon Pang, Hursuong Vongsachang, Thomas K. Le, George Q. Zhang, Taibo Li, Jason T. C. Lee, Shari M. Lawson

**Affiliations:** 1 Johns Hopkins University School of Medicine, 733 N. Broadway, Miller Research Building 137, Baltimore, MD 21205 USA; 2 Department of Gynecology and Obstetrics, Johns Hopkins University School of Medicine, 733 N. Broadway, Miller Research Building 137, Baltimore, MD 21205 USA

**Keywords:** Cultural competency, Cultural humility, Undergraduate medical education, Asian/Asian American patients

## Abstract

**Background:**

Asian Americans (AsAm) are a rapidly growing population in the U.S. With this growing population, U.S. healthcare providers must be equipped to provide culturally competent care for AsAm patients. This project surveyed U.S. medical students on their knowledge of and attitudes towards AsAm to assess predictors of readiness to care for AsAm patients.

**Method:**

This cross-sectional study surveyed medical students who had completed at least one clinical rotation. The survey was distributed online to nine medical schools throughout the U.S. The survey measured self-rated knowledge of, comfort with, cultural competency (CC) towards, and explicit biases towards AsAm patients. The first three domains were analyzed in a multivariate regression model including sociodemographic characteristics and past clinical, curricular, and social experiences with AsAm. Explicit bias questions were reported descriptively.

**Results:**

There were 688 respondents. Asian race, AsAm-prevalent hometown, AsAm-related extracurricular activities, Asian language knowledge, and having taken a population health course predicted increased AsAm knowledge. Social interactions with AsAm increased comfort with AsAm patients. Increasing year in medical school, more frequent exposure to AsAm patients on rotations, and prior travel to an Asian country were predictors of increased CC toward AsAm. Importantly, having completed a CC course was a significant predictor in all domains. In terms of explicit bias, students felt that AsAm patients were more compliant than Caucasian patients. Students also believed that Caucasian patients were generally more likely to receive self-perceived “preferred” versus “acceptable” care, but that in their own clinical experiences neither group received preferred care.

**Conclusion:**

Experience with and exposure to AsAm prior to and during medical school and CC courses may increase medical student knowledge, comfort, and CC with AsAm patients. Standardized and longitudinal CC training, increased simulations with AsAm patients, diverse student recruitment, and support for students to engage in AsAm-related activities and interact with AsAm may improve CC of future physicians towards AsAm patients and possibly other minority populations.

**Supplementary Information:**

The online version contains supplementary material available at 10.1186/s12909-021-02568-0.

## Background

Cultural competency (CC) is an important skill in providing quality healthcare for diverse patient populations. Defined as behaviors, attitudes, and policies that enable effective cross-cultural work [1], culturally competent practices in the clinical setting have been associated with positive healthcare experiences, particularly for minorities [2, 3]. Asian Americans (AsAms) are the fastest growing minority group in the U.S. [4] and are predicted to become the nation’s largest immigrant group by 2065 [5]. With this growing population, U.S. healthcare providers must be equipped to provide culturally competent care for AsAm patients. Previous studies have shown that AsAm patients report fewer positive interactions within healthcare and lack of cultural understanding by their physicians [6, 7]. Additionally, foreign-born AsAm patients have among the poorest self-reported health [8], potentially due to low health literacy, language barriers, and cultural factors complicating healthcare delivery [9–12]. Such factors can lead to decreased satisfaction with care and fear of approaching the healthcare system [13].

CC training often begins in medical school, and is a requirement for medical student competencies and accreditation [14, 15]. However, despite efforts at implementation [1, 16], gaps still remain in undergraduate medical CC education. In previous studies, students reported feeling uncomfortable and unprepared to approach culture-related clinical issues and did not view understanding diverse beliefs as important in delivering effective care [17, 18]. Formal CC training is often limited to a few lectures during the pre-clinical curriculum with minimal cross-cultural training or practical experience during rotations [18–20].

Studies on CC training have been conducted in both undergraduate [21] and graduate [22] medical education, and for Black [23], Latino [24], and gender and sexual minority [25] populations. However, few studies have investigated CC among medical students as it relates to AsAm patients. Given the overall lack of research examining provider perceptions towards AsAm patients and the impact on care [26], more research is needed to assess predictors of medical student CC for AsAm patients [27–29]. The present survey assesses students’ perceived knowledge, attitudes, and skills on caring for the AsAm population, as well as explicit biases towards this population. In doing so, we aim to identify factors influencing medical students’ perceptions of and readiness to take care of AsAm patients in the U.S.

## Methods

### Survey design

A cross-sectional survey was adapted with permission from the Medical and Nursing Student Readiness to Treat Latino Patients (MaNSRT©) survey developed and tested by Mayo and Sherrill in 2014 [24], with additional questions on explicit bias adapted from Sabin et al. [30]. We adapted questions from the MaNSRT© survey by substituting “Latino” in the question stems with “Asian and/or Asian American” and added questions assessing additional experiential predictors. Explicit bias questions were adapted by substituting “Asian Americans and/or Asians” and “Caucasian” for “African Americans” and “White/European Americans”, respectively in the original question stems. After these modifications were made, the survey was pilot-tested with small groups of medical students to assess for understanding and modified appropriately. This study received approval from the Institutional Review Board at the Johns Hopkins School of Medicine (IRB00201533) and support from a Josiah Macy Jr. Foundation President’s Grant and a Dr. Elizabeth Small Grant from the Johns Hopkins School of Medicine.

While more recent literature has leaned towards using the terms “cultural humility” to indicate these skills and attitudes as an ongoing pursuit [31, 32] and “adherent to therapy” rather than “compliant”, our survey used the terms “cultural competency” (CC) [24] and “compliant” [30] as adopted from the original surveys, in order to minimize deviation from the language used in the original surveys.

### Data collection

The survey was distributed online via Qualtrics XM (Qualtrics, Provo, UT) to nine medical schools across the U.S. through each school’s preferred distribution channel (e.g. email lists) from September 2019 to May 2020. Schools were selected through convenience sampling based upon existing student networks with the intent of having geographical representation. A current medical student at each school sent the initial recruitment email, with a reminder email 2 weeks later. Surveyed schools included Baylor, David Geffen at UCLA, Dell, Harvard, Johns Hopkins, Medical College of Wisconsin, Northwestern Feinberg, Stanford, and Washington University in St. Louis. Upon completing the anonymous survey, respondents could enter their email address to receive a $5 Amazon gift card. The survey recruitment email specified that only students who completed at least one clinical rotation were invited to participate and detailed the risks and benefits of participating. All respondents provided written consent by completing the survey.

Sociodemographic factors assessed include age, gender, race/ethnicity (non-Hispanic White, Asian, Black, Hispanic, Other or Multicultural), medical school, year in medical school (M1/2, M3, M4, research year/alternative degree), and ZIP code of “hometown” if in the U.S. Percent Asian population in hometown was identified by asking each respondent to self-identify their 5-digit hometown ZIP code, and then corresponding that 5-digit ZIP code to total population as well as Asian alone counts using the American Community Survey 2018 5-year estimates [33]. Percentages were then grouped by quartile (1st quartile: least to 4th quartile: most). International medical students who reported hometowns without a U.S. ZIP code were excluded from this analysis. The survey assessed personal experiential factors including frequency of interaction with AsAm populations and exposure to AsAm topics self-rated on a Likert scale, and binary questions on life experiences pertaining to AsAm exposure.

Primary outcomes include self-reported experience in three main domains: knowledge of AsAm patients, comfort in treating AsAm patients, and CC towards AsAm patients (5, 5, and 8 questions respectively). Self-reported explicit bias towards AsAm patients was assessed separately (6 questions). Questions were presented in Likert scale format (Additional file 2). Responses for the three main domains were each reported as an average composite score from 1 to 5, with higher scores indicating higher or more positive self-reported experience.

### Internal validity

To assess the internal validity of survey questions across knowledge, comfort, and CC domains, we first performed principal component analysis on dependent variables and observed clustering of questions from the same category on the top two principal components (53 and 9% of variance respectively). We then constructed a confirmatory factor analysis model on three correlated factors, resulting in a reasonable fit (comparative fit index: 0.80; root mean square error of approximation: 0.12; standardized root mean square residual: 0.09).

### Statistics

Sociodemographic/experiential factors and explicit bias were tabulated and reported as frequencies and percentages. Mean composite scores by domain were reported as means and standard deviations. Univariable and multivariable linear regression models were used to examine the association between predictors and each of the composite domain scores. Covariates were included in adjusted models at a threshold of *p* < 0.25. Kruskal-Wallis rank sum test was used to identify associations between respondent race and hometown AsAm population with self-reported warmth towards AsAm patients. All statistical analyses were conducted in either R 3.4.0 (R Foundation for Statistical Computing, Vienna, Austria) or Stata/MP 15.1 (StataCorp LP, College Station, TX). Statistical significance was defined at *p* < 0.05.

## Results

### Study cohort

Of 883 surveys, we excluded 59 (6.7%) that were incomplete and 136 (15.4%) based on ineligibility. A total of 688 surveys were included for analysis (Table 1). Response rate across all schools was approximately 23% (range:11–49%), based on available information on class sizes and duration of clinical curriculum.

**Table 1:** Sociodemographic characteristics of cohort (*N* = 688)

Sociodemographic Characteristics	N	%
Age, years (Median, IQR)	25, 24–27	
Gender		
Female	355	51.6
Male	330	48.0
Non-binary/Prefer not to answer	3	0.4
Race/Ethnicity		
Asian	284	41.3
Non-Hispanic White	258	37.5
Hispanic	31	4.5
Black	27	3.9
Other/Multiracial	88	12.8
Among Asians (*N* = 319)		
Chinese	122	38.2
Indian	80	25.1
Korean	28	8.8
Vietnamese	12	3.8
Filipino	10	3.1
Japanese	9	2.8
Pakistani	5	1.6
Indonesian	3	0.9
Malaysian	1	0.3
Prefer not to say	4	1.25
Other/Multiracial	45	14.1
Medical School		
Harvard	160	23.3
Johns Hopkins	118	17.2
Medical College of Wisconsin	94	13.7
Washington University in St. Louis	91	13.2
David Geffen at UCLA	61	8.9
Baylor	56	8.14
Northwestern Feinberg	48	7.0
Stanford	43	6.3
Dell	17	2.5
Year in Medical School		
M1/M2	63	9.2
M3	306	44.5
M4	227	33.0
Other (Other degree, year off, etc.)	92	13.4
% Asians in Hometown (Median, IQR)	7.2, 3.2–15.5	
% Asians in Hometown, by cohort %ile		
0–3.2% (1st quartile)	157	22.8
3.2–7.2% (2nd)	160	23.3
7.2–15.5% (3rd)	161	23.4
15.5–74.6% (4th)	164	23.8
Did not answer	46	6.7

The median age of all participants was 25 years (IQR: 24–27). Participants were distributed evenly between males (48.0%, *N* = 330) and females (51.6%, *N* = 355). Most participants identified as either Asian only (41.3%, *N* = 284) or non-Hispanic White only (37.5%, *N* = 258). The composition of survey respondents overrepresented AsAm medical students for certain schools, but not others (Supplementary Table 1). Harvard, Johns Hopkins, and Medical College of Wisconsin composed 54.2% (*N* = 372) of respondents. A majority were third- (44.5%, *N* = 306) or fourth-year students (33.0%, *N* = 227). Nine percent (9.2%, *N* = 63) were first- and second-years, and 13.4% (*N* = 92) were pursuing an alternative opportunity during their medical program (e.g., research year, alternative degree). The majority reported a U.S. hometown (93.3%, *N* = 642). Median population percentage of AsAm individuals in participants’ hometown was 7.2% (IQR: 3.2–15.5%).

**Fig. 1:**
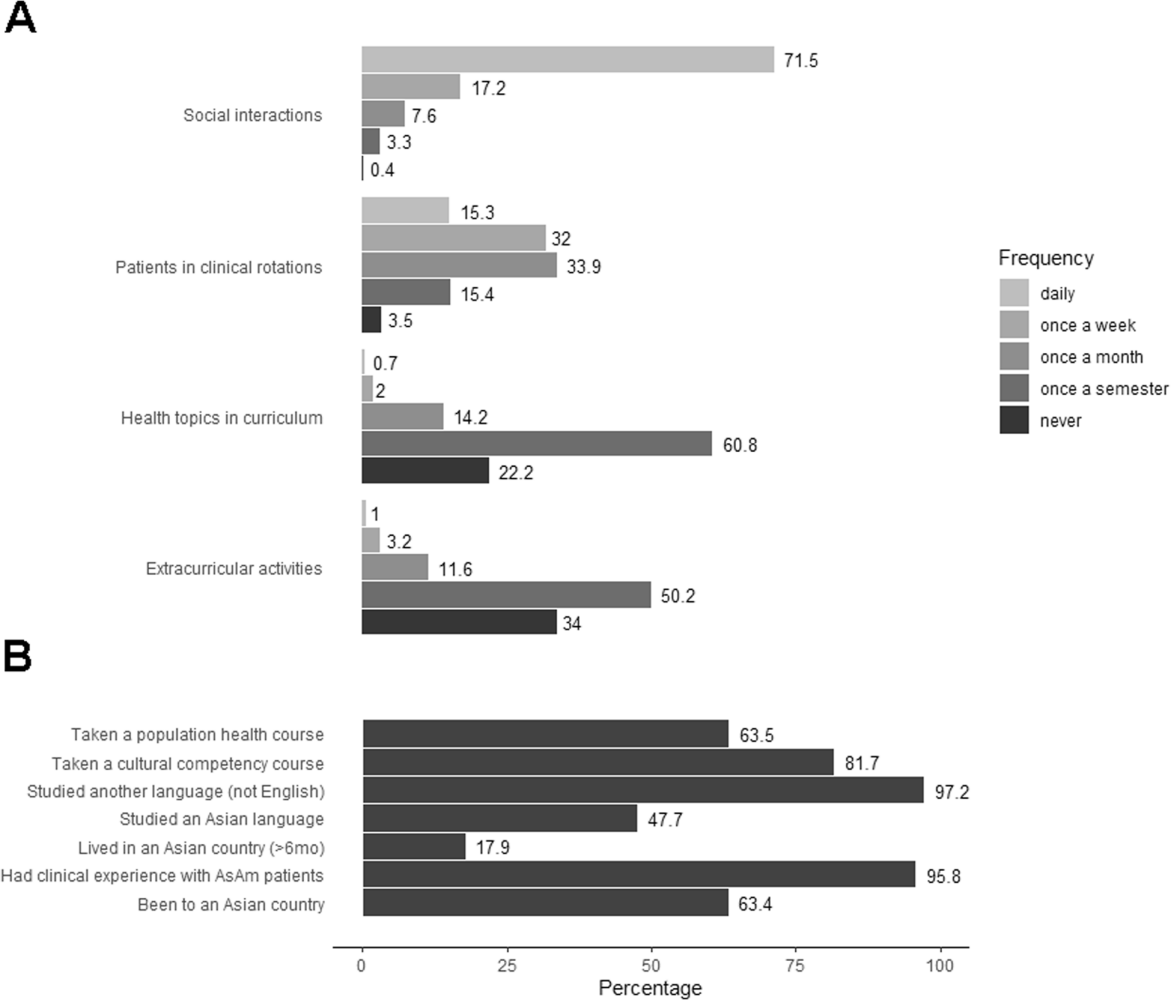
Self-reported Asian American (AsAm)-related experiences of cohort (*N* = 688). **a** Frequency of AsAm-related experiences. **b** Binary questions on current and past experiences (“yes” or “no”)

The majority of medical students reported never or once or less per semester participating in activities focused on AsAm populations (84.2%, *N* = 579) and gaining exposure to AsAm health topics in the curriculum (82.8%, *N* = 569) (Fig. 1). Most students reported having taken a CC course (81.7%, *N* = 562) or population health course (63.5%, *N* = 436). The majority of students reported clinical experience with AsAm patients (95.8%, *N* = 659), with 32% (*N* = 220) reporting exposure to AsAm patients on clinical rotations once a week and 33.9% (*N* = 233) reporting once a month. The majority (71.5%, *N* = 491) reported daily social interactions with AsAm persons in the past year. Most students (63.4%, *N* = 436) had visited an Asian country and 17.9% (*N* = 123) lived in an Asian country for more than 6 months. The vast majority of students reported having studied another language (97.2%, *N* = 668); 47.7% (*N* = 328) had studied an Asian language.

### Knowledge of AsAm patients

The mean score for AsAm knowledge across the survey cohort was 2.80 out of 5 (SD = 0.84) (Supplementary Table 2). After adjusting for baseline differences in sociodemographic and experience factors, CC course completion was independently associated with increased reported knowledge about AsAm patients (b = 0.17, 95% CI [0.02, 0.31]) (Table 2). Asian race (vs. White race, b = 0.41, 95% CI [0.22, 0.59]), growing up in a hometown with the highest quartile of Asian population (vs. lowest quartile, b = 0.18, 95% CI [0.00, 0.35]), increasing participation in AsAm-focused extracurricular activities (b = 0.18, 95% CI [0.10, 0.25]), having studied an Asian language (b = 0.33, 95% CI [0.17, 0.49]), and having taken a population health course (b = 0.12, 95% CI [0.01, 0.24]) also remained significantly associated with increased knowledge of AsAm patients.

**Table 2:** Adjusted analysis of factors associated with primary domains^a^

	Knowledge		Comfort		Cultural Competency	
Sociodemographic Characteristics	Beta (95% CI)	p	Beta (95% CI)	p	Beta (95% CI)	p
Age (Years)	–		0.01 (− 0.01, 0.04)	0.214	–	
Gender						
Male	–		Ref		Ref	
Female, non-binary, no answer	–		0.08 (−0.01, 0.18)	0.095	−0.09 (− 0.18, 0.01)	0.053
Race/Ethnicity						
White	Ref		Ref		Ref	
Asian	0.41 (0.22, 0.59)	< 0.001	− 0.08 (− 0.20, 0.05)	0.223	− 0.07 (− 0.20, 0.06)	0.289
Hispanic	− 0.09 (− 0.35, 0.18)	0.510	− 0.09 (− 0.32, 0.14)	0.456	0.01 (− 0.21, 0.23)	0.924
Black	− 0.125 (− 0.41, 0.16)	0.387	− 0.29 (− 0.59, − 0.00)	0.049	− 0.15 (− 0.38, 0.09)	0.228
Other (incl. multiracial)	0.14 (− 0.03, 0.31)	0.116	− 0.05 (− 0.20, 0.10)	0.529	− 0.03 (− 0.17, 0.11)	0.692
Year in Medical School						
M1/M2	Ref		Ref		Ref	
M3	− 0.06 (− 0.27, 0.16)	0.597	0.10 (− 0.09, 0.29)	0.293	0.18 (0.00, 0.36)	0.048
M4	− 0.04 (− 0.26, 0.18)	0.721	0.01 (− 0.20, 0.21)	0.951	0.27 (0.09, 0.45)	0.003
Other (i.e., year off, etc.)	0.18 (−0.22, 0.25)	0.882	0.08 (−0.14, 0.29)	0.487	0.12 (−0.08, 0.31)	0.249
Medical school						
Johns Hopkins	Ref		Ref		Ref	
Harvard	−0.22 (−0.39, − 0.04)	0.016	− 0.05 (− 0.21, 0.10)	0.499	0.22 (0.08, 0.37)	0.003
Medical College of Wisconsin	−0.06 (− 0.26, 0.14)	0.548	0.08 (− 0.10, 0.26)	0.397	0.04 (− 0.13, 0.20)	0.676
Wash. U. in St. Louis	0.07 (−0.13, 0.27)	0.478	0.09 (−0.08, 0.27)	0.287	0.22 (0.05, 0.38)	0.010
David Geffen at UCLA	0.10 (−0.13, 0.32)	0.400	0.06 (−0.13, 0.26)	0.538	0.26 (0.08, 0.45)	0.005
Baylor	0.03 (−0.21, 0.27)	0.800	0.05 (−0.16, 0.25)	0.664	0.32 (0.12, 0.51)	0.002
Northwestern Feinberg	0.03 (−0.21, 0.27)	0.793	−0.05 (− 0.25, 0.16)	0.656	0.21 (0.01, 0.41)	0.037
Stanford	−0.12 (− 0.38, 0.13)	0.345	− 0.15 (− 0.37, 0.07)	0.180	−0.01 (− 0.23, 0.20)	0.898
Dell	−0.13 (− 0.49, 0.24)	0.492	0.04 (− 0.28, 0.35)	0.828	0.51 (0.20, 0.81)	0.001
% Asians in Hometown, by %ile						
1st quartile	Ref		–		–	
2nd	0.04 (−0.12, 0.19)	0.643	–		–	
3rd	0.00 (−0.16, 0.16)	0.974	–		–	
4th	0.18 (0.00, 0.35)	0.044	–		–	
How often have you gained exposure in AsAm-related...						
Extracurricular activities	0.18 (0.10, 0.25)	< 0.001	−0.02 (−0.08, 0.04)	0.608	0.06 (−0.00, 0.12)	0.060
Health topics in curriculum	–		–		0.03 (−0.04, 0.10)	0.393
Patients in clinical rotations	−0.01 (− 0.06, 0.05)	0.859	0.05 (− 0.01, 0.10)	0.095	0.07 (0.02, 0.12)	0.008
Social interactions	–		0.09 (0.03, 0.16)	0.005	0.04 (−0.02, 0.10)	0.171
Have you ever...						
Been to an Asian country	0.13 (−0.01, 0.26)	0.065	–		0.11 (0.00, 0.23)	0.048
Lived in an Asian country (>6mo)	0.08 (−0.07, 0.24)	0.306	–		–	
Studied an Asian language	0.33 (0.17, 0.49)	< 0.001	–		–	
Studied other language	−0.06 (−0.40, 0.28)	0.743	0.27 (−0.04, 0.58)	0.091	–	
Taken a population health course	0.12 (0.01, 0.24)	0.033	–		0.06 (−0.04, 0.15)	0.234
Taken a cultural competency course	0.17 (0.02, 0.31)	0.023	0.15 (0.03, 0.28)	0.027	0.13 (0.01, 0.25)	0.033
Had clinical experience with AsAm pts	0.19 (−0.09, 0.47)	0.180	0.09 (−0.15, 0.33)	0.480	0.04 (−0.20, 0.27)	0.761

^a^Predictors with *p* < 0.25 on unadjusted analysis were included in the adjusted model

Abbreviation: *AsAm* Asian American

### Comfort with AsAm patients

The mean score for comfort with AsAm patients was 4.42 out of 5 (SD = 0.56) (Supplementary Table 2). Adjusted multivariable linear regression demonstrated that CC course completion (b = 0.15, 95% CI [0.03, 0.28]) and increasing social interactions with AsAm in the past year (b = 0.09, 95% CI [0.03, 0.16]) were independently associated with increased reported comfort with AsAm patients (Table 2).

### Cultural competency towards AsAm patients

The mean score for CC towards AsAm patients was 3.78 out of 5 (SD = 0.60) (Supplementary Table 2). In adjusted models, CC course completion remained an independent predictor of CC towards AsAm patients (b = 0.13, 95% CI [0.01, 0.25]) (Table 2). Other significant predictors included being a third-year (vs. first- and second-year, b = 0.18, 95% CI [0.00, 0.36]) and fourth-year student (vs. first- and second-year, b = 0.27, 95% CI [0.09, 0.45]), increasing exposure with AsAm patients on clinical rotations (b = 0.07, 95% CI [0.02, 0.12]), and having been to an Asian country (b = 0.11, 95% CI [0.00, 0.23]).

### Explicit bias

Median warmth towards both AsAm and Caucasians were 6 out of 7 (1: coldest - 7: warmest). The majority of students showed no difference for either group (61.1%, *N* = 378), while 32.1% (*N* = 199) reported warmer feelings towards AsAm compared to Caucasians. Asian race and higher hometown AsAm population were associated with more warmth towards AsAm patients (*p* < 0.001 for both).

On average, students reported a neutral feeling for relative compliance (median score of 4) between AsAm and Caucasian patients both from general perception and their own clinical experiences (1: AsAm more likely – 7: Caucasians more likely) (Table 3). More than 1/3 of participants reported that AsAm patients are more compliant (general perception, 38.5%, *N* = 185; clinical experiences, 40.0%, *N* = 180), compared to 16.5% (*N* = 79) and 23.5% (*N* = 93) who reported Caucasian patients as more compliant based on their general perception and clinical experiences, respectively. The majority of students reported that from their perspective, Caucasian patients (74.7%, *N* = 384) were more likely to receive preferred versus acceptable care than AsAm patients (4.7%, *N* = 24) in general. However, in their own clinical experiences, 67.6% (*N* = 267) reported no difference in which group receives preferred care.

**Table 3:** Self-reported attitudes toward race and medical care^a^

	AsAm More Likely		Both Groups Equally Likely		Caucasian More Likely	
	N	[%]	N	[%]	N	[%]
My feelings towards AsAm/Caucasians are warm^b^. (*N* = 619)	199	32.1	378	61.1	42	6.8
In general, which group is more compliant? (*N* = 480)	185	38.5	216	45.0	79	16.5
In your clinical experience, which group is more compliant? (*N* = 451)	180	40.0	189	41.9	82	18.2
In general, which group receives preferred care? (*N* = 514)	24	4.7	106	20.6	384	74.7
In your clinical experience, which group receives preferred care? (*N* = 395)	35	8.9	267	67.6	93	23.5

^a^N varies for each question as respondents (N = 688) had a chance to indicate “Do not know”

^b^This result is generated from respondents who answered both questions about warmth towards AsAm and Caucasians

Abbreviation: *AsAm* Asian American

## Discussion

This study assessed factors representing the four major components of CC: awareness of biases, attitudes, knowledge, and skills [31]. In our multivariable regression model, predictors of increased AsAm knowledge included Asian race, AsAm-prevalent hometown, AsAm-related extracurricular activities, Asian language knowledge, and population health course completion. Increasing social interactions with AsAm predicted increasing comfort with AsAm patients. Finally, third- and fourth-year status, more frequent exposure to AsAm patients on rotations, and prior travel to an Asian country were predictors of increasing CC skills towards AsAm patients. Notably, CC course completion was a significant predictor for all three domains.

While our results suggested that medical students may increase their knowledge of Asian values and belief systems regarding health by investing time in relevant opportunities, it may be impractical to implement AsAm-centric language classes or extracurricular activities across all medical schools. Encouraging or assessing activities that demonstrate interest in diverse cultures among medical school applicants could be an avenue to increase AsAm knowledge among future medical school cohorts. Our results also suggested that population health courses served as an opportunity to increase AsAm knowledge, perhaps by focusing on AsAm-specific health topics. More efforts are needed to understand how to create effective learning opportunities regarding AsAm knowledge within medical school curricula.

We found that AsAm heritage and AsAm-prevalent hometown predicted increased self-rated AsAm knowledge, suggesting a need for greater AsAm diversity in medical school recruitment to promote a culturally knowledgeable workforce to care for various AsAm populations [34]. The composition of AsAm subgroups in medical schools do not reflect that of the U.S. population. For example, despite Filipinos being the second largest AsAm subgroup in the U.S. [4], they only represent 4.3% of all AsAm medical school applicants and even fewer AsAm matriculants [35]. Strategies to recruit medical students of specific AsAm subgroups are needed so that future physicians may bring their unique cultural backgrounds to patient care.

Frequent social interactions with AsAm being a predictor of increased comfort suggests that casual exposure to AsAm can help students develop skills that translate clinically. Nearly 30% of respondents reported socially interacting with AsAm once per week or fewer times in the past year. The aforementioned strategy of diverse recruitment of AsAm medical students can provide students with peer interactions to build confidence and comfort with AsAm populations.

More frequent exposure to AsAm patients on rotations and travel to an Asian country predicted greater self-reported CC, suggesting that meaningful and immersive interaction with AsAm populations may be beneficial for developing CC skills. Ensuring optimal clinical opportunities with AsAm patients is necessary to facilitate CC training. In this study cohort, nearly 20% of students reported that their exposure to AsAm patients was once a semester or less, or never at all. Schools with relatively smaller AsAm patient populations can increase simulations and vignettes with AsAm patients. Simulations, such as virtual or standardized patient encounters, are effective in providing CC training [36–38]. Schools can also support away rotations in AsAm-dense regions or international experiences. Interestingly, AsAm race was not a significant predictor of comfort or CC, while factors indicating AsAm exposure and experience were, suggesting that comfort and CC skills can be learned. Third-, and to a larger degree, fourth-year status were also a predictor of increased CC, consistent with the idea that these skills improve with experience.

Having taken a CC course was consistently associated with higher knowledge, comfort, and CC. Prior work has established the effectiveness of CC training for healthcare professionals [27], further supporting that CC skills can be acquired through training in medical school. While CC education is part of the core medical school curriculum, its implementation—including structure/format, content, and requirements—varies widely across programs. Furthermore, standardized implementation guidelines and educator resources are lacking [36, 38]. Currently, the Association of American Medical Colleges provides a 67-item checklist for medical schools to assess the topics covered in their CC curriculum, without further guidance regarding implementation [39]. Despite being mandatory, nearly 20% of respondents reported never having taken a CC course; secondary analysis demonstrated no difference by school. Respondents possibly did not feel that their coursework constituted a “CC course” or adequately provided any applicable skills. This finding, in addition to higher CC scores in fourth years and the positive association between CC course completion and scores in all domains, pushes for the development of an effective, standardized, and longitudinal CC curriculum throughout medical school.

To the best of our knowledge, our study is the only other application of this survey instrument beyond the original Sherrill et al. study assessing student perceptions of Latino patients [24]. While both studies found that language proficiency predicted knowledge, race congruency was also predictive in our study. The higher proportion of Asian respondents (41.3%) in our study versus 4.7% Hispanic respondents in the Sherrill study could contribute to this. Hometown, year in school, extracurricular activities, CC course completion, and the frequency of social interactions and clinical exposure to the population of interest, all significant predictors in our study, were not included in the Sherrill study. Finally, their findings that social interactions with Latinos in the past year, prior experience living in a Spanish-speaking country, and Spanish language proficiency predicted CC could be related to differences in respondent demographics and composition between the two studies, variation in scoring, and different effects of prior experiences on CC towards Latino versus AsAm patients.

We also sought to survey the explicit attitudes of medical students towards AsAm patients, as bias has been shown to affect physician decision making and patient outcomes [40]. Our study respondents reported no particular distinction in warmth towards either AsAm or White patients or to whom they would deliver preferred care, which may be partially explained by social desirability biases [41]. However, prior surveys of medical students have found that the absence of explicit differences in warmth does not mark the absence of implicit bias or bias in decision-making [42]. Meanwhile, respondents reported that White patients generally received preferred care, and that AsAm patients were more compliant, nearly double than those who reported White patients as more compliant. In other populations, compliance stereotyping and bias have been shown to negatively affect patient encounters, including poor ratings of care, poor visit communication, and biased clinical decision making [30, 43, 44]. However, it is difficult to fully extrapolate similar conclusions towards the treatment of AsAm patients, as few literature exists for this population in this area [40]. Despite this limitation, the presence of previously unreported explicit biases for AsAm patients warrants further investigation, including how biases may affect clinical practice and strategies to reduce bias during medical training.

There are several limitations to our study. The cross-sectional design limits the interpretation of causation. Additionally, the survey collected self-reported rather than measured knowledge, comfort, bias, and CC, limiting our understanding of respondents’ true knowledge, attitudes, and skills by self-report, social desirability biases, and overestimation [45, 46]. As noted earlier, we decided to use the terms “cultural competence” and “compliant” rather than “cultural humility” and “adherent,” respectively, which are more current and accepted, in order to minimize variation from the original surveys from which our survey was adopted, as well as to more directly compare our results with prior literature that used these older terms. However, we intend to use more current terminology in our future work.

Another important limitation of our study is the general nature of the survey questions directed towards AsAm as an aggregate group. Because of this, we were unable to specify results towards specific subgroups in our analysis, although medical students may have varied perceptions and experiences with different subgroups. We recognize that AsAm are a highly diverse population with unique sociocultural considerations and health disparities among subgroups and the lack of disaggregation of the AsAm population in the current literature [47]. However, because our research goal was to obtain a national sample of medical student perceptions towards AsAm and conduct an exploratory investigation within this area, we chose to focus on examining perceptions of AsAm generally, which also allowed us to distribute a more concise and digestible survey. While this choice limits the application of these results to various AsAm subgroups, we hope that this study demonstrates the need for further work distinguishing medical students’ perceptions towards different AsAm subgroups. Future work can build on the findings of this survey by starting with specifying medical student knowledge, attitudes, and experiences with the largest AsAm subgroups in the U.S.

Our survey generated many responses across schools with different geographies and demographics and accounted for differences between schools in the models. However, the convenience sampling method at a limited number of U.S. medical schools and low overall response rate at 23% of eligible students at the surveyed schools, despite incentives and email reminders, limited true generalizability. Differences in school size and schools’ distribution methods may explain the variation in school response numbers and rates. Further, AsAm students were over-represented in our sample and White, Black, and Hispanic students were under-represented with respect to U.S. medical student demographics [48]. To address this bias, we adjusted for race in our model and compared explicit bias responses between Asian and non-Asian medical students. Finally, while our survey was based on a previously developed and tested survey [24], our confirmatory factor analysis suggested a moderate model fit, which could possibly be explained by adapting a survey designed originally for Latino patients to AsAm patients and variations in how medical students perceive different racial groups. More work is needed to develop a survey for AsAm patients.

## Conclusions

To our knowledge, this study is the first nation-wide distribution of surveys assessing medical students’ perceived CC and explicit biases towards AsAm patients. Our study found that 1) experience with and exposure to AsAm populations and topics prior to and during medical school and CC course completion were associated with increased medical student self-reported knowledge, comfort, and CC with AsAm patients, and 2) medical students self-reported biases towards AsAm patients compared to White patients. Given the relative lack of CC research for this population, we believe these findings can inform curricular reform and enhance medical school CC training for AsAm and other minority populations, as well as inspire further research in this area. Future directions include validating a survey designed specifically to assess knowledge and attitudes of medical students toward AsAm subgroups and/or conducting student focus groups, investigating implicit biases towards AsAm patients among U.S. physicians, and implementing and evaluating interventions. Through these efforts, medical students will be better equipped to serve the growing AsAm population as future physicians.

## Supplementary Information

**Additional file 1: Supplementary Table S1.** Percentage of Students Identifying as Asian American in Study Cohort and Overall Class^a^, by Medical School. **Supplementary Table S2.** Mean composite scores (1–5), by domain.

**Additional file 2:** Survey questions.

## Data Availability

The datasets used and/or analysed during the current study are available from the corresponding author on reasonable request.

## Abbreviations

CC: cultural competency
AsAm: Asian American(s)

## References

[1] Jernigan VBB, Hearod JB, Tran K, Norris KC, Buchwald D (2016). An examination of cultural competence training in US medical education guided by the tool for assessing cultural competence training. J Health Disparities Res Pract.

[2] Weech-Maldonado R, Elliott M, Pradhan R, Schiller C, Hall A, Hays RD (2012). Can hospital cultural competency reduce disparities in patient experiences with care?. Med Care.

[3] Mygind A, Norredam M, Nielsen AS, Bagger J, Krasnik A (2008). The effect of patient origin and relevance of contact on patient and caregiver satisfaction in the emergency room. Scand J Public Health.

[4] Hoeffel EM, Rastogi S, Kim MO, Shahid H. The Asian Population: 2010 [Internet]. 2012 Mar [cited 2020 May 21]. Available from: https://www.census.gov/prod/cen2010/briefs/c2010br-11.pdf

[5] López G, Ruiz NG, Patten E (2017). Key facts about Asian Americans, a diverse and growing population [internet]. Pew research center.

[6] Taira DA, Safran DG, Seto TB, Rogers WH, Inui TS, Montgomery J (2001). Do patient assessments of primary care differ by patient ethnicity?. Health Serv Res.

[7] Ngo-Metzger Q, Legedza ATR, Phillips RS (2004). Asian Americans’ reports of their health care experiences. Results of a national survey. J Gen Intern Med.

[8] Du Y, Xu Q (2016). Health disparities and delayed health care among older adults in California: a perspective from race, ethnicity, and immigration. Public Health Nurs Boston Mass.

[9] Mora N, Golden SH (2017). Understanding cultural influences on dietary habits in Asian, middle eastern, and Latino patients with type 2 diabetes: a review of current literature and future directions. Curr Diab Rep.

[10] Sentell T, Braun KL (2012). Low health literacy, limited English proficiency, and health status in Asians, Latinos, and other racial/ethnic groups in California. J Health Commun.

[11] Lee HY, Rhee TG, Kim NK, Ahluwalia JS (2015). Health literacy as a social determinant of health in Asian American immigrants: findings from a population-based survey in California. J Gen Intern Med.

[12] Sorkin DH, Ngo-Metzger Q (2014). The unique health status and health care experiences of older Asian Americans: research findings and treatment recommendations. Clin Gerontol.

[13] Shepherd SM, Willis-Esqueda C, Paradies Y, Sivasubramaniam D, Sherwood J, Brockie T (2018). Racial and cultural minority experiences and perceptions of health care provision in a mid-western region. Int J Equity Health.

[14] The Core Competencies for Entering Medical Students [Internet]. Association of American Medical Colleges; [cited 2020 May 21]. Available from: https://students-residents.aamc.org/applying-medical-school/article/core-competencies/

[15] Functions and Structure of a Medical School: Accreditation and the Liaison Committee on Medical Education. Liaison Committee on Medical Education; 2020.

[16] Lanting K, Dogra N, Hendrickx K, Nathan Y, Sim J, Suurmond J. Culturally Competent in Medical Education – European Medical Teachers’ Self-Reported Preparedness and Training Needs to Teach Cultural Competence Topics and to Teach a Diverse Class. MedEdPublish [Internet]. 2019 [cited 2020 Jun 29];8(2). Available from: https://www.mededpublish.org/manuscripts/2188 10.15694/mep.2019.000098.1 PMC10712631 38089273

[17] Loue S, Wilson-Delfosse A, Limbach K (2015). Identifying gaps in the cultural competence/sensitivity components of an undergraduate medical school curriculum: a needs assessment. J Immigr Minor Health.

[18] Green AR, Chun MBJ, Cervantes MC, Nudel JD, Duong JV, Krupat E (2017). Measuring medical students’ preparedness and skills to provide cross-cultural care. Health Equity.

[19] Flores G, Gee D, Kastner B (2000). The teaching of cultural issues in U.S. and Canadian medical schools. Acad Med J Assoc Am Med Coll.

[20] Kripalani S, Bussey-Jones J, Katz MG, Genao I (2006). A prescription for cultural competence in medical education. J Gen Intern Med.

[21] Seeleman C, Hermans J, Lamkaddem M, Suurmond J, Stronks K, Essink-Bot M-L (2014). A students’ survey of cultural competence as a basis for identifying gaps in the medical curriculum. BMC Med Educ.

[22] Chun MBJ, Yamada A-M, Huh J, Hew C, Tasaka S (2010). Using the cross-cultural care survey to assess cultural competency in graduate medical education. J Grad Med Educ.

[23] van Ryn M, Hardeman R, Phelan SM, PhD DJB, Dovidio JF, Herrin J (2015). Medical school experiences associated with change in implicit racial Bias among 3547 students: a medical student CHANGES study report. J Gen Intern Med.

[24] Sherrill WW, Mayo RM, Truong KD, Pribonic AP, Schalkoff CA (2016). Assessing medical student cultural competence: what really matters. Int J Med Educ.

[25] Phelan SM, Burke SE, Hardeman RR, White RO, Przedworski J, Dovidio JF (2017). Medical school factors associated with Changes in implicit and explicit Bias against gay and lesbian people among 3492 graduating medical students. J Gen Intern Med.

[26] Ho IK, Lawrence JS. The role of social cognition in medical decision making with Asian American patients. J Racial Ethn Health Disparities. 2020 Sep;14. 10.1007/s40615-020-00867-8 PMC7489188 32926390

[27] Beach MC, Price EG, Gary TL, Robinson KA, Gozu A, Palacio A (2005). Cultural competence: a systematic review of health care provider educational interventions. Med Care.

[28] Loudon RF, Anderson PM, Gill PS, Greenfield SM (1999). Educating medical students for work in culturally diverse societies. JAMA..

[29] Brach C, Fraser I (2000). Can cultural competency reduce racial and ethnic health disparities? A review and conceptual model. Med Care Res Rev MCRR.

[30] Sabin JA, Rivara FP, Greenwald AG (2008). Physician implicit attitudes and stereotypes about race and quality of medical care. Med Care.

[31] Tervalon M, Murray-García J (1998). Cultural humility versus cultural competence: a critical distinction in defining physician training outcomes in multicultural education. J Health Care Poor Underserved.

[32] Greene-Moton E, Minkler M (2020). Cultural competence or cultural humility? Moving beyond the debate. Health Promot Pract.

[33] 2018 American Community Survey 5-year estimates. Table B02001. [Internet]. US Census Bureau; 2018 [cited 2020 Jun 15]. Available from: https://data.census.gov/cedsci/

[34] Zou P (2017). Diet and blood pressure control in Chinese Canadians: cultural considerations. J Immigr Minor Health.

[35] Figure 3. Percentage of Asian (alone) applicants to U.S. medical schools by Asian subgroups, academic year 2018-2019 | AAMC [Internet]. Association of American Medical Colleges; [cited 2020 May 21]. Available from: https://www.aamc.org/data-reports/workforce/interactive-data/figure-3-percentage-asian-alone-applicants-us-medical-schools-asian-subgroups-academic-year-2018

[36] Brottman MR, Char DM, Hattori RA, Heeb R, Taff SD (2020). Toward cultural competency in health care: a scoping review of the diversity and inclusion education literature. Acad Med J Assoc Am Med Coll..

[37] Zhang C, Cho K, Chu J, Yang J (2014). Bridging the gap: enhancing cultural competence of medical students through online videos. J Immigr Minor Health.

[38] Deliz JR, Fears FF, Jones KE, Tobat J, Char D, Ross WR (2020). Cultural competency interventions during medical school: a scoping review and narrative synthesis. J Gen Intern Med.

[39] Tool for Assessing Cultural Competence Training (TACCT) | AAMC [Internet]. Association of American Medical Colleges; [cited 2020 May 21]. Available from: https://www.aamc.org/what-we-do/mission-areas/diversity-inclusion/tool-for-assessing-cultural-competence-training

[40] Hall WJ, Chapman MV, Lee KM, Merino YM, Thomas TW, Payne BK (2015). Implicit racial/ethnic Bias among health care professionals and its influence on health care outcomes: a systematic review. Am J Public Health.

[41] Steenkamp J-BEM, De Jong MG, Baumgartner H (2010). Socially desirable response tendencies in survey research. J Mark Res.

[42] Cormack D, Harris R, Stanley J, Lacey C, Jones R, Curtis E (2018). Ethnic bias amongst medical students in Aotearoa/New Zealand: findings from the Bias and decision making in medicine (BDMM) study. PLoS One.

[43] Oliver MN, Wells KM, Joy-Gaba JA, Hawkins CB, Nosek BA (2014). Do physicians’ implicit views of African Americans affect clinical decision making?. J Am Board Fam Med JABFM.

[44] Cooper LA, Roter DL, Carson KA, Beach MC, Sabin JA, Greenwald AG (2012). The associations of clinicians’ implicit attitudes about race with medical visit communication and patient ratings of interpersonal care. Am J Public Health.

[45] Katowa-Mukwato P, Banda S (2016). Self-perceived versus objectively measured competence in performing clinical practical procedures by final year medical students. Int J Med Educ.

[46] Sawdon M, Finn G (2014). The “unskilled and unaware” effect is linear in a real-world setting. J Anat.

[47] Yi SS, Kwon SC, Sacks R, Trinh-Shevrin C (2016). Commentary: persistence and health-related consequences of the model minority stereotype for Asian Americans. Ethn Dis.

[48] Table B-5.2: Total Enrollment by U.S. Medical School and Race/Ethnicity (Alone or In Combination), 2019–2020 [Internet]. Available from: https://www.aamc.org/system/files/2019-11/2019_FACTS_Table_B-5.2.pdf

