# Supplementary material for: Knowledge and attitudes of U.S. medical students regarding the care of Asian American patients: a cross-sectional survey study
Source: BMC Med Educ. 2021 Mar 6;21:148. doi: 10.1186/s12909-021-02568-0 (PMC7937206; doi:10.1186/s12909-021-02568-0)
Supplement: Supplementary file 1 — Additional file 1: Supplementary Table S1. Percentage of Students Identifying as Asian American in Study Cohort and Overall Classa, by Medical School. Supplementary Table S2. Mean composite scores (1–5), by domain. [file 12909_2021_2568_MOESM1_ESM.docx]

**Supplementary Table 1.** Percentage of Students Identifying as Asian American in Study Cohort and Overall Class^a^, by Medical School.

| Medical School | % Asian American Among Study Cohort | % Asian American Among Overall Class |
| --- | --- | --- |
| Harvard | 41.9 | 26.5 |
| Johns Hopkins | 39.8 | 34.8 |
| Medical College of Wisconsin | 12.8 | 14.0 |
| Washington University in St. Louis | 44.0 | 28.3 |
| David Geffen at UCLA | 55.7 | 36.2 |
| Baylor | 58.9 | 36.6 |
| Northwestern Feinberg | 56.3 | 36.5 |
| Stanford | 48.8 | 35.3 |
| Dell | 17.7 | 20.2 |

^a^Data retrieved from: Association of American Medical Colleges. 2020 FACTS: Enrollment, Graduates, and MD-PhD Data. Published November 5, 2019. <https://www.aamc.org/data-reports/students-residents/interactive-data/2020-facts-enrollment-graduates-and-md-phd-data>.

**Supplementary Table 2.** Mean composite scores (1-5), by domain.^a^

| **Sociodemographic characteristics** | **Knowledge**  Mean (SD) | **Comfort**  Mean (SD) | **Cultural Competency**  Mean (SD) |
| --- | --- | --- | --- |
| **Overall:** | 2.80 (0.84) | 4.42 (0.56) | 3.78 (0.60) |
| Gender |  |  |  |
| Male | 2.78 (0.88) | 4.37 (0.61) | 3.81 (0.62) |
| Female, non-binary, no answer | 2.82 (0.81) | 4.47 (0.51) | 3.75 (0.58) |
| Race/Ethnicity |  |  |  |
| White | 2.41 (0.65) | 4.50 (0.50) | 3.79 (0.57) |
| Asian | 3.31 (0.77) | 4.36 (0.60) | 3.77 (0.61) |
| Hispanic | 2.35 (0.60) | 4.43 (0.53) | 3.82 (0.55) |
| Black | 2.26 (0.78) | 4.23 (0.62) | 3.60 (0.68) |
| Other (including multiracial) | 2.66 (0.81) | 4.43 (0.58) | 3.79 (0.66) |
| Year in Medical School |  |  |  |
| M1/M2 | 2.62 (0.88) | 4.21 (0.74) | 3.63 (0.59) |
| M3 | 2.80 (0.87) | 4.47 (0.50) | 3.78 (0.56) |
| M4 | 2.85 (0.82) | 4.40 (0.60) | 3.85 (0.64) |
| Other (Other degree, year off, etc.) | 2.83 (0.76) | 4.44 (0.49) | 3.67 (0.58) |
| Medical school |  |  |  |
| Harvard | 2.68 (0.78) | 4.33 (0.64) | 3.79 (0.58) |
| Johns Hopkins | 2.87 (0.86) | 4.39 (0.61) | 3.64 (065) |
| Medical College of Wisconsin | 2.45 (0.69) | 4.47 (0.48) | 3.61 (0.58) |
| Washington University in St. Louis | 2.92 (0.88) | 4.54 (0.54) | 3.84 (0.57) |
| David Geffen at UCLA | 3.11 (0.88) | 4.51 (0.47) | 3.92 (0.59) |
| Baylor | 2.93 (0.87) | 4.49 (0.47) | 3.91 (0.58) |
| Northwestern Feinberg | 3.05 (0.93) | 4.39 (0.50) | 3.87 (0.53) |
| Stanford | 2.93 (0.75) | 4.36 (0.59) | 3.74 (0.63) |
| Dell | 2.39 (0.78) | 4.43 (0.45) | 4.04 (0.54) |
| % Asians in Hometown, by cohort %ile |  |  |  |
| 1st quartile | 2.51 (0.78) | 4.42 (0.56) | 3.76 (0.59) |
| 2nd | 2.66 (0.83) | 4.48 (0.51) | 3.77 (0.60) |
| 3rd | 2.74 (0.82) | 4.41 (0.54) | 3.75 (0.61) |
| 4th | 3.22 (0.80) | 4.38 (0.63) | 3.82 (0.61) |

^a^On a scale from 1-5, 1 is the lowest and 5 is the highest.
