# Supplementary material for: Knowledge and attitudes of U.S. medical students regarding the care of Asian American patients: a cross-sectional survey study
Source: BMC Med Educ. 2021 Mar 6;21:148. doi: 10.1186/s12909-021-02568-0 (PMC7937206; doi:10.1186/s12909-021-02568-0)
Supplement: Supplementary file 2 [file 12909_2021_2568_MOESM2_ESM.docx]

Knowledge and Attitudes of U.S. Medical Students Regarding the Care of Asian American Patients

Start of Block: SURVEY INSTRUCTIONS

Start of Block: Student Resources

Q1 
We are a group of medical students from Johns Hopkins who are hoping to assess gaps in cultural competency medical education with an emphasis on Asian American patients. The Asian American population is the fastest growing minority group in the US, with a 46% growth between 2000-2010 (Hoeffel, Rastogi, Kim & Shahid 2012). Studies demonstrate the need for health care providers to become more culturally competent, but there is no published data to date assessing physicians and medical students perceived cultural competency specifically in the Asian American population. Access to healthcare is an important consideration in public health policy, but delivery to immigrant and minority groups are markedly complicated by unique cultural values and practices, as well as low English fluency and literacy levels.

 

Please note that participation in this study is entirely voluntary and responses will be de-identified and kept confidential. Participation or declining participation in this study will have absolutely no impact on any of your grades or evaluations. There are no known risks related to participation in the study beyond what you may encounter in everyday life. **At the end of the survey, you will have the chance to leave your email to receive a $5 Amazon gift card and be entered into a raffle for several Apple watches as tokens of our appreciation.**

This survey will take approximately 5-10 minutes to complete. You may stop the survey at any time. 

 

Your completion of the survey or questionnaire will serve as your consent to be in this research study.

 

If you have any questions about the study, please contact Shari Lawson at slawson2@jhmi.edu 

 

Thank you for helping us learn about the current status of medical training with regards to the Asian American population and how we can advocate for our patients. 

 

(PI Shari Lawson, MD MBA, Johns Hopkins IRB00201533)

 

 

Q2 What is your age?

|  | 0 | 10 | 20 | 30 | 40 | 50 | 60 | 70 | 80 | 90 | 100 |
| --- | --- | --- | --- | --- | --- | --- | --- | --- | --- | --- | --- |

| Age in years () |  |
| --- | --- |

Q3 Gender

- Female  (1) 

- Male  (2) 

- Nonbinary  (3) 

- I prefer not to answer  (4) 

Q4 What is your race or ethnicity? (please select all that apply):  

 

- White  (23) 

- Hispanic, Latino, or Spanish  (24) 

- Black or African American  (25) 

- Asian  (26) 

- American Indian or Alaskan Native  (27) 

- Middle Eastern or North African  (28) 

- Native Hawaiian or Other Pacific Islander  (29) 

- Not sure or don't know  (30) 

- Some other race or ethnicity (please specify)  (31) ________________________________________________

Display This Question:

If What is your race or ethnicity? (please select all that apply):     = Asian

|  |
| --- |

Q5 If you identify yourself as Asian, which of the following apply to you? (please select all that apply)

- Chinese  (241) 

- Indian  (242) 

- Filipino  (243) 

- Vietnamese  (244) 

- Korean  (245) 

- Japanese  (246) 

- Pakistani  (247) 

- Cambodian  (248) 

- Hmong  (249) 

- Malaysian  (250) 

- Indonesian  (251) 

- I prefer not to answer  (252) 

- Other (please specify)  (253) ________________________________________________

Q6 What is the zip code of your hometown? If you've lived in multiple locations, please select the location that you consider most formative to your overall development. For international places, please enter city name and country. 

________________________________________________________________

Q7 Which medical school do you attend? 

▼ Johns Hopkins University School of Medicine (4) ... David Geffen School of Medicine at UCLA (12)

Q8 Which year of medical school are you in?

- M1  (97) 

- M2  (98) 

- M3  (99) 

- M4  (100) 

- Currently pursuing another degree (e.g. MPH, PhD)  (101) 

- Currently taking a research year(s)  (103) 

- Other (please specify)  (102) ________________________________________________

Q9 Have you completed at least one clinical rotation?

- Yes  (10) 

- No  (11) 

Skip To: End of Survey If Have you completed at least one clinical rotation? = No

Q10 Have you encountered Asian and/or Asian American patients on your clinical rotations? 

- Yes  (1) 

- No  (2) 

- Not sure  (3) 

Q11 
Please note that "Asian" refers to any or all of the following categories: South Asian (for example Indian), East Asian (for example Chinese), and Southeast Asian (for example Vietnamese).

 
How often do you feel you have... 

|  | Never (1) | Once a semester or less (2) | Once a month (3) | Once a week (4) | Daily (5) |
| --- | --- | --- | --- | --- | --- |
| participated in one or more extracurricular activities with a focus on an Asian and/or Asian American population? (1) |  |  |  |  |  |
| gained exposure to Asian and/or Asian American health topics in your medical school curriculum? (2) |  |  |  |  |  |
| gained exposure to Asian and/or Asian American patients in clinical rotations in medical school? (3) |  |  |  |  |  |
| had social interactions with Asian and/or Asian Americans in the past year? (4) |  |  |  |  |  |

Q12 
Have you...

|  | No (8) | Yes (9) | Don't remember or don't know (10) |
| --- | --- | --- | --- |
| been to an Asian country? (366) |  |  |  |
| lived in an Asian country (for longer than six months)? (367) |  |  |  |
| ever learned or studied an Asian language? (368) |  |  |  |
| ever learned or studied a language other than English? (371) |  |  |  |
| ever taken a population health course? (372) |  |  |  |
| ever taken a course that covered cultural competency for patients? (373) |  |  |  |
| had some clinical experience with Asian and/or Asian American patients? (374) |  |  |  |

| Page Break |  |
| --- | --- |

Q13 
Please rate the next two statements from 1-7, where 1=cold and 7=warm. 

 

My feelings towards **Caucasians** are... 

|  | cold | warm |
| --- | --- | --- |

|  | 1 | 2 | 3 | 4 | 5 | 6 | 7 |
| --- | --- | --- | --- | --- | --- | --- | --- |

| 1 () |  |
| --- | --- |

Q14 

Please note that "Asian" refers to any or all of the following categories: South Asian (for example Indian), East Asian (for example Chinese), and Southeast Asian (for example Vietnamese). 

 

My feelings towards **Asian Americans and/or Asians** are... (1=cold and 7=warm)

|  | cold | warm |
| --- | --- | --- |

|  | 1 | 2 | 3 | 4 | 5 | 6 | 7 |
| --- | --- | --- | --- | --- | --- | --- | --- |

| 1 () |  |
| --- | --- |

| Page Break |  |
| --- | --- |

Q15 
We would like you to continue rating the next set of four statements from 1-7, where 

 

1 = Asians or Asian Americans (AA) are more likely

4 = Asians or Asian Americans (AA) and Caucasians are equally likely

7 = Caucasians are more likely

 
**In general,** members of which group are more likely to be compliant patients?

|  | Asians or AAs are more likely | Asians or AAs and Caucasians are equally likely | Caucasians are more likely | Don't know or N/A |
| --- | --- | --- | --- | --- |

|  | 1 | 2 | 3 | 4 | 5 | 6 | 7 |
| --- | --- | --- | --- | --- | --- | --- | --- |

| 1 () |  |
| --- | --- |

Q16 
**In your own clinical experience**, members from which group are more likely be compliant patients?

|  | Asians or AAs are more likely | Asians or AAs and Caucasians are equally likely | Caucasians are more likely | Don't know or N/A |
| --- | --- | --- | --- | --- |

|  | 1 | 2 | 3 | 4 | 5 | 6 | 7 |
| --- | --- | --- | --- | --- | --- | --- | --- |

| 1 () |  |
| --- | --- |

Q17 **In general**, which group is more likely to receive preferred medical care versus acceptable medical care?

|  | Asians or AAs are more likely | Asians or AAs and Caucasians are equally likely | Caucasians are more likely | Don't know or N/A |
| --- | --- | --- | --- | --- |

|  | 1 | 2 | 3 | 4 | 5 | 6 | 7 |
| --- | --- | --- | --- | --- | --- | --- | --- |

| 1 () |  |
| --- | --- |

Q18 In your own clinical experience, for which group are you more likely to prescribe preferred ​medical care versus acceptable medical care?

|  | Asians or AAs are more likely | Asians or AAs and Caucasians are equally likely | Caucasians are more likely | Don't know or N/A |
| --- | --- | --- | --- | --- |

|  | 1 | 2 | 3 | 4 | 5 | 6 | 7 |
| --- | --- | --- | --- | --- | --- | --- | --- |

| 1 () |  |
| --- | --- |

| Page Break |  |
| --- | --- |

Q19 
Based on your training and experience, please rate your comfort level with your skills in the following aspects of care for Asian and/or Asian American patients.

|  | Not at all comfortable (1) | Not very comfortable (2) | Somewhat comfortable (3) | Very comfortable (4) | Extremely comfortable (5) |
| --- | --- | --- | --- | --- | --- |
| Determining patients' communication preferences (1) |  |  |  |  |  |
| Taking social history (2) |  |  |  |  |  |
| Assessing patients' understanding of the cause of their illnesses (3) |  |  |  |  |  |
| Explaining the treatment plan to the patient (4) |  |  |  |  |  |
| Identifying how well a patient can read or write in English (5) |  |  |  |  |  |
| Identifying religious beliefs that might impact clinical care (6) |  |  |  |  |  |
| Identifying cultural (non-religious) beliefs that might impact clinical care (7) |  |  |  |  |  |
| Using a translator to communicate (9) |  |  |  |  |  |

Q20 
In your opinion, how often...

|  | Almost never (1) | Less than half the time (6) | About half the time (2) | More than half the time (3) | Almost always (4) | Not applicable (7) |
| --- | --- | --- | --- | --- | --- | --- |
| do you avoid speaking to Asian or Asian American patients because of language barriers when providing clinical care? (1) |  |  |  |  |  |  |
| are you able to explain treatment options and clinical trials to Asian or Asian American patients? (2) |  |  |  |  |  |  |
| do you feel you are able to build satisfactory rapport with Asian or Asian American  patients? (3) |  |  |  |  |  |  |
| are you less satisfied with Asian patients than non-Asian patients? (4) |  |  |  |  |  |  |
| do you approach the treatment of Asian or Asian American patients differently than non-Asian or non-Asian American patients? (5) |  |  |  |  |  |  |

Q21 
How familiar are you with the values and belief systems for Asian or Asian American patients related to...

|  | Not at all familiar (1) | Not very familiar (2) | Somewhat familiar (3) | Very familiar (4) | Extremely familiar (5) |
| --- | --- | --- | --- | --- | --- |
| Health (1) |  |  |  |  |  |
| Illness (2) |  |  |  |  |  |
| Help-seeking behaviors (3) |  |  |  |  |  |
| Traditional healing practices (4) |  |  |  |  |  |
| Preference for traditional healers (5) |  |  |  |  |  |

Q22 
(Optional) Please note that "Asian" refers to any or all of the following categories: South Asian (for example Indian), East Asian (for example Chinese), and Southeast Asian (for example Vietnamese).

 
How much more do you want to learn about Asian and Asian American health disparities and topics specific to these patients? Any other thoughts about your preparation to care for these patients? 

________________________________________________________________

________________________________________________________________

________________________________________________________________

________________________________________________________________

________________________________________________________________

Q25 **If you would like to receive a $5 Amazon gift card and be entered into a raffle for Apple watches, please enter your email below. Your email will only be used for the purposes of the gift card and raffle, and not for anything else. Please use your .edu email address.**

________________________________________________________________

End of Block: Student Resources
